# Intranasally Applied Neuropeptide S Shifts a High-Anxiety Electrophysiological Endophenotype in the Ventral Hippocampus towards a "Normal"-Anxiety One

**DOI:** 10.1371/journal.pone.0120272

**Published:** 2015-04-01

**Authors:** Julien Dine, Irina A. Ionescu, Charilaos Avrabos, Yi-Chun Yen, Florian Holsboer, Rainer Landgraf, Ulrike Schmidt, Matthias Eder

**Affiliations:** 1 Max Planck Institute of Psychiatry, Munich, Germany; 2 Department Stress Neurobiology and Neurogenetics, Max Planck Institute of Psychiatry, Munich, Germany; The Chinese University of Hong Kong, HONG KONG

## Abstract

The neurobiological basis of pathological anxiety and the improvement of its pharmacological treatment are a matter of intensive investigation. Here, using electrophysiological techniques in brain slices from animals of the high anxiety-related behavior (HAB) and normal anxiety-related behavior (NAB) mouse model, we show that basal neurotransmission at ventral hippocampal CA3-CA1 synapses is weaker in HAB compared to NAB mice. We further demonstrate that paired-pulse facilitation (PPF) and long-term potentiation (LTP) at these synapses are more pronounced in slices from HAB animals. Based on previous findings, we also examined whether intranasal delivery of neuropeptide S (NPS), which increasingly emerges as a potential novel treatment option for anxiety symptoms occurring in a variety of diseases like anxiety disorders, posttraumatic stress disorder, and major depression, impacts on the high-anxiety electrophysiological endophenotype in HAB mice. Strikingly, we detected enhanced basal neurotransmission and reduced PPF and LTP in slices from NPS-treated HAB animals. Collectively, our study uncovers a multifaceted high-anxiety neurophysiological endophenotype in the murine ventral hippocampus and provides the first evidence that an intranasally applied neuropeptide can shift such an endophenotype in an anxiety-regulating brain structure towards a “normal”-anxiety one.

## Introduction

Anxiety symptoms occur in various psychiatric diseases, such as anxiety and depressive disorders [1] and schizophrenia [2], yet the neurophysiological basis of pathological anxiety remains poorly understood and improved pharmacotherapies are vitally needed [3–4]. A brain structure which in the last decade turned out to be involved in the regulation of anxiety-related behaviors in mammals is the ventral hippocampus (vHPC) [5–10] and a recent study has unveiled a significant contribution of excitatory synaptic transmission onto CA1 pyramidal neurons to this process [11]. It is therefore highly interesting to investigate the modulation of physiological properties of the vHPC by anxiolytic substances in mouse models of anxiety disorders. We have previously investigated modulatory effects of neuropeptide S (NPS), a potential novel treatment option for pathological anxiety [12–15], in the vHPC [13]. In mice, NPS precursor mRNA is found in only two brain regions: the Kölliker-Fuse nucleus and the pericoerulear area [16]. NPS-producing neurons can be activated *inter alia* by stress [17] and corticotropin-releasing factor [18] and project to regions involved in the anxiety-related circuitry such as the basolateral amygdala [16], where presynaptically located NPS receptors (NPSR) modulate glutamate release and anxiety [12]. NPSR, the only cognate NPS receptor, is a G protein-coupled receptor (GPCR) coupled to either G_q_ or G_s_ [19] and widely expressed in the brain, including anxiety-related regions [16]. We previously demonstrated in mice that NPS decreases anxiety-related behavior upon microinjection into area CA1 of the vHPC [20]. Moreover, we showed that intranasally applied NPS, which exerts anxiolytic-like effects, expands into the vHPC [13]. Based on these facts and findings, we investigated here whether hyper- and “normally”-anxious mice [21] exhibit differences in neurotransmission and/or plasticity at ventral CA3-CA1 synapses and, since we detected such differences, whether intranasally administered NPS impacts on the high-anxiety endophenotype. In particular, we analyzed basal neurotransmission, paired-pulse facilitation (PPF), and long-term potentiation (LTP) at these synapses in brain slices from animals of the well-validated high anxiety-related behavior (HAB) and normal anxiety-related behavior (NAB) mouse model [21–23]. HAB mice display pathologically high levels of anxiety-like behaviors compared to NAB counterparts. The elevated plus maze (EPM) was selected as an initial evaluation tool, but HAB animals also exhibit elevated anxiety-like behavior in the dark-light test as well as fewer signs of risk assessment and higher ultrasound vocalization calls, all measures being an indication of high-anxiety levels [21]. In addition, we performed identical electrophysiological analyses in vHPC slices from HAB and NAB mice which were treated intranasally with NPS, to investigate for the first time whether vHPC electrophysiological properties are influenced by NPS in a similar way in pathological and normal anxiety states.

## Materials and Methods

### Animals

For all experiments, male mice were used. HAB mice, representing CD-1 mice which have been selectively and bidirectionally inbred for high anxiety-related behavior on the EPM (>40 generations) [21], were obtained from the animal facilities of the Max Planck Institute of Psychiatry (MPIP, Munich, Germany). “Normally” anxious wild-type CD-1 (NAB) mice were also bred in the animal facilities of the MPIP [21], [23]. Animals were kept in cages of four with food and water *ad libitum*, in a 12 h dark-light cycle. Behavioral testing on the EPM was performed in 7-week-old animals which afterwards were separated in 2 cohorts (HAB and NAB) and kept until use for electrophysiological recordings at the age of 12 weeks. Experiments were approved by the Committee on Animal Health and Care of the government of Upper Bavaria and conducted in compliance with the guidelines for the care and use of laboratory animals set by the European Union Directive 86/609/EEC.

*Behavioral testing* of animals on the EPM was carried out on postnatal day 49 as described by Krömer et al. [21]. Weight for HAB mice: 30 ± 0.35 g; weight for NAB mice: 30 ± 2 g. The EPM test served to select the animals according to their anxiety levels (high anxiety *vs*. “normal” anxiety). The test was performed during the light cycle and lasted 5 min. Animals were placed on the EPM facing the closed arm. The lighting levels were adjusted to 300 lux in the open arms, 30–50 lux in the neutral zone, and 5–10 lux in the closed arms. The maze was cleaned after each animal using odorless soap and water, and subsequently dried.

*Intranasal administration of NPS* was performed as we described previously [13], [20]. Briefly, alert mice, previously habituated to handling, were restrained manually in a supine position during the administration procedure, with the head immobile at an angle of approximately 45° to the body. 7 *μ*l NPS (7 nmol)-containing artificial cerebrospinal fluid (ACSF) or 7 *μ*l pure ACSF (Vehicle (Veh), for composition see below) were pipetted alternatingly to each nostril without touching the nasal mucosa; after 5 min, the procedure was repeated, for a total application of 14 nmol of NPS or vehicle. Afterwards, animals were allowed to rest for 2 h before preparation of brain slices.

### Preparation of brain slices

12-week-old mice were anesthetized with isoflurane and decapitated. All following steps were done in ice-cold cutting saline saturated with carbogen gas (95% O_2_/5% CO_2_). This saline (pH 7.4) contained (in mM): 125 NaCl, 2.5 KCl, 25 NaHCO_3_, 1.25 NaH_2_PO_4_, 0.5 CaCl_2_, 6 MgCl_2_, and 25 glucose. After decapitation, the brain was rapidly removed from the cranial cavity and 350-*μ*m-thick horizontal slices containing the vHPC were cut using a vibratome (HM650V, Thermo Scientific). Subsequently, slices were incubated for 30 min in carbogenated ACSF at 34°C. The ACSF (pH 7.4) consisted of (in mM): 125 NaCl, 2.5 KCl, 25 NaHCO_3_, 1.25 NaH_2_PO_4_, 2 CaCl_2_, 1 MgCl_2_, and 25 glucose. Afterwards, slices were stored at room temperature (23–25°C) for at least 90 min in carbogenated ACSF before electrophysiological measurements.

### Electrophysiology

All animals were coded and experiments performed in blind (comparison HAB *vs*. NAB; HAB Veh, HAB NPS, NAB Veh, and NAB NPS). The code was broken after analysis of the data. All experiments were performed at room temperature. Only the first two to three slices from the ventral surface of the brain in which the CA1 region was clearly visible were used for the measurements. In the recording chamber, slices were continuously superfused with carbogenated ACSF (4–5 ml/min flow rate). Neurotransmission at CA3-CA1 synapses was evoked by square pulse electrical stimuli (50 *μ*s pulse width, 0.066 Hz in the LTP experiments) delivered via a custom-made bipolar tungsten electrode (50 *μ*m pole diameter, ∼0.5 MΩ nominal impedance) to the Schaffer collateral-commissural pathway. The resultant excitatory postsynaptic potentials (EPSPs) were extracellularly recorded using glass microelectrodes (filled with ACSF, ~1 MΩ open-tip resistance) which were placed into the CA1 stratum radiatum. For the LTP and PPF measurements, the intensity of voltage stimulation was adjusted in a manner to produce a field EPSP (fEPSP) of ∼50% of the amplitude at which a population spike appeared. Recording data were low-pass filtered at 500 Hz and digitized at 2.5 kHz. LTP was induced by high-frequency stimulation (HFS, 100 Hz for 1 s). The paired-pulse ratio was calculated as fEPSP2 amplitude/fEPSP1 amplitude. In every slice, we first conducted “input-output” measurements at CA3-CA1 synapses and afterwards assessed PPF and LTP.

### Chemicals

Rat NPS was purchased from Bachem (Bubendorf, Switzerland). The salts for the cutting saline and the ACSF were from Sigma-Aldrich (Taufkirchen, Germany) and isoflurane from Abbott (Wiesbaden, Germany).

### Statistics

Statistical analysis was performed using Sigma Stat 3.5 and GraphPad Prism 5.03. Statistical significance was assessed by means of the two-tailed unpaired Student's *t*-test or a one-, two- or three-way repeated measures ANOVA, followed by Tukey *post hoc* tests, if appropriate. Data are given as mean ± SEM with significance declared at *p <* 0.05. In all graphs, p values are depicted as follows: ★*p* < 0.05, ★★*p* < 0.01, ★★★*p* < 0.001; n.s., not statistically significant.

## Results

### HAB and NAB mice exhibit differences in basal neurotransmission, PPF, and LTP at ventral CA3-CA1 synapses

**Fig 1:**
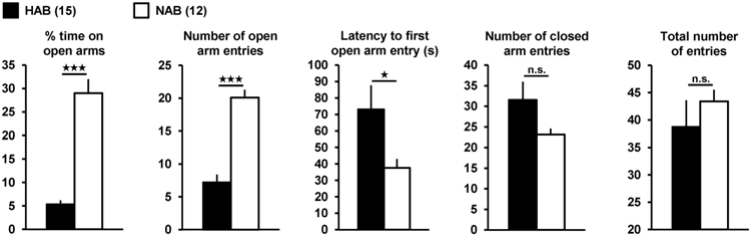
EPM parameters of the HAB (*n* = 15) and NAB (*n* = 12) mice used for the electrophysiological experiments shown in Fig. 2. EPM testing time was 5 min.

To potentially uncover a high-anxiety neurophysiological endophenotype in the murine vHPC, we performed field potential measurements of basal neurotransmission, PPF, and LTP at ventral CA3-CA1 synapses in brain slices from HAB and NAB mice. EPM tests confirmed that the HAB and NAB animals under study exhibited exaggerated and “normal” anxiety-related behavior [21], [23] (Fig. 1; HAB (*n* = 15) *vs*. NAB (*n* = 12): % time on open arms: *t*_(25)_ = -8.1, *p* < 0.001; number of open arm entries: *t*_(25)_ = -7.3, *p* < 0.001; latency to first open arm entry: *t*_(25)_ = 2.1, *p* = 0.043; number of closed arm entries: *t*_(25)_ = 1.6, *p* = 0.118; total number of entries: *t*_(25)_ = -0.8, *p* = 0.425; two-tailed unpaired *t*-tests with 25 degrees of freedom).

**Fig 2:**
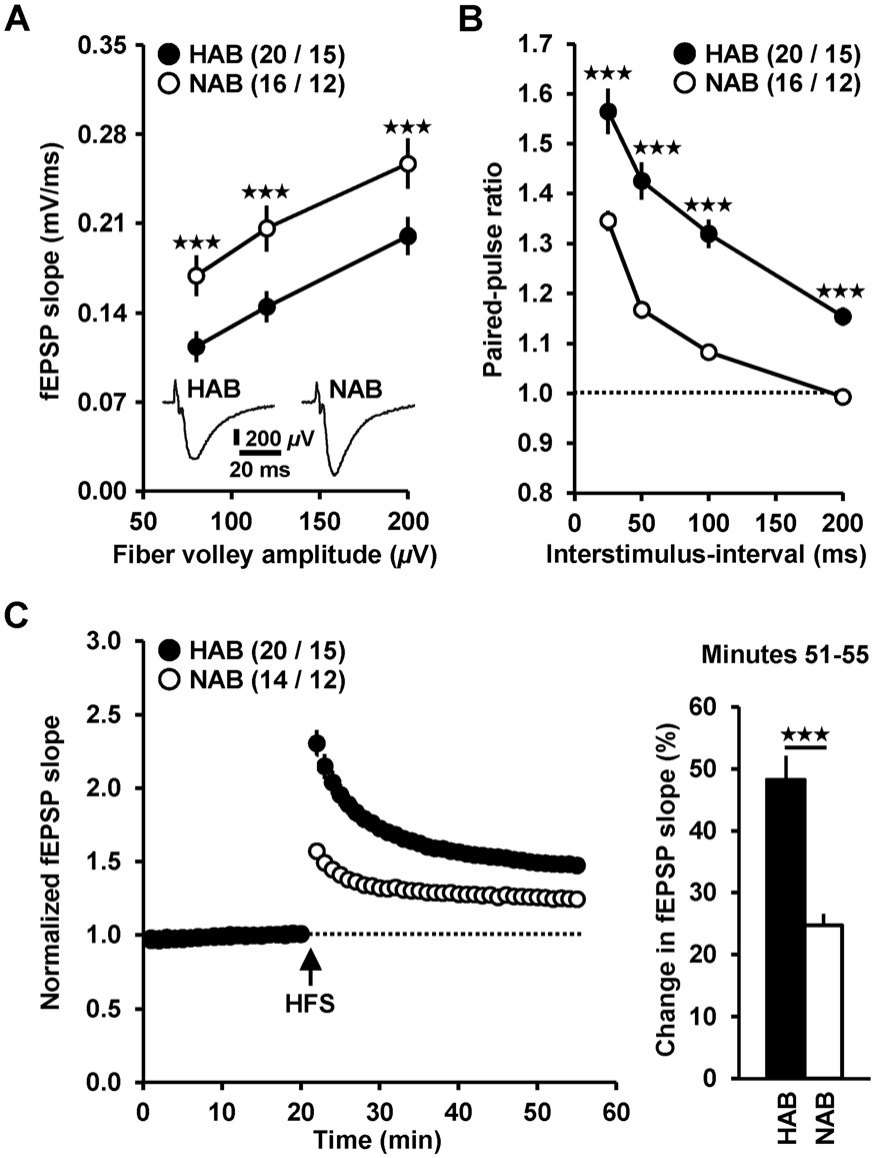
HAB (black circles) and NAB (open circles) mice exhibit differences in electrophysiological properties in the vHPC. (*A*) HAB mice show weaker basal neurotransmission since the input-output curve is shifted towards smaller fEPSP amplitudes compared to NAB animals. (*B*) PPF is stronger in HAB compared to NAB animals at interstimulus-intervals of 25, 50, 100, and 200 ms, suggesting a lower probability of neurotransmitter release. (*C*) LTP at ventral CA3-CA1 synapses is more pronounced in slices from HAB mice. For clarity, stimulus artifacts in recording traces (*A*) were truncated in part. *n* numbers are indicated in brackets as follows (slices / animals).

Strikingly, we detected differences in all neurophysiological parameters tested. As evident from the input-output curves depicted in Fig. 2A, basal neurotransmission was weaker in slices from HAB mice than in those obtained from NAB animals (HAB (*n* = 20 slices / 15 mice) *vs*. NAB (*n* = 16 slices / 12 mice): two-way repeated measures ANOVA: Phenotype: F_1,61_ = 7.822, *p* = 0.009; Fiber volley amplitude (FVA): F_2,61_ = 314.055, *p* < 0.001; Phenotype x FVA: F_2,61_ = 1.084, *p* = 0.345; Tukey *post hoc* tests HAB *vs*. NAB, FVA 80, 120, 200 *μ*V: *p* < 0.001).

In contrast, PPF and LTP were more pronounced in slices prepared from HAB mice (Fig. 2*B*-*C*; PPF: two-way repeated measures ANOVA: Phenotype: F_1,102_ = 32.612, *p* < 0.001; Interstimulus-interval (ISI): F_3,102_ = 264.634, *p* < 0.001; Phenotype x ISI: F_3,102_ = 6.592, *p* < 0.001; Tukey *post hoc* tests HAB *vs*. NAB, ISI 25, 50, 100, 200: *p* < 0.001; LTP minutes 51–55: two-tailed unpaired *t*-test: *t*_(32)_ = 4.7, *p* < 0.001).

### Intranasally applied NPS causes enhanced basal neurotransmission and reduced PPF and LTP at ventral CA3-CA1 synapses in HAB mice

Next, we investigated whether intranasally administered NPS impacts on the high-anxiety endophenotype in HAB mice and, if so, shifts it towards a “normal”-anxiety one. EPM tests confirmed that the animals under study exhibited high anxiety-related behavior (data not shown). The electrophysiological measurements were conducted 4–5 h after NPS or Veh treatment of HAB and NAB mice. We chose this time period since NPS was previously shown to lead to an anxiolytic-like effect in HAB or C57Bl/6N mice 4–5 h after intranasal application [13], [20].

**Fig 3:**
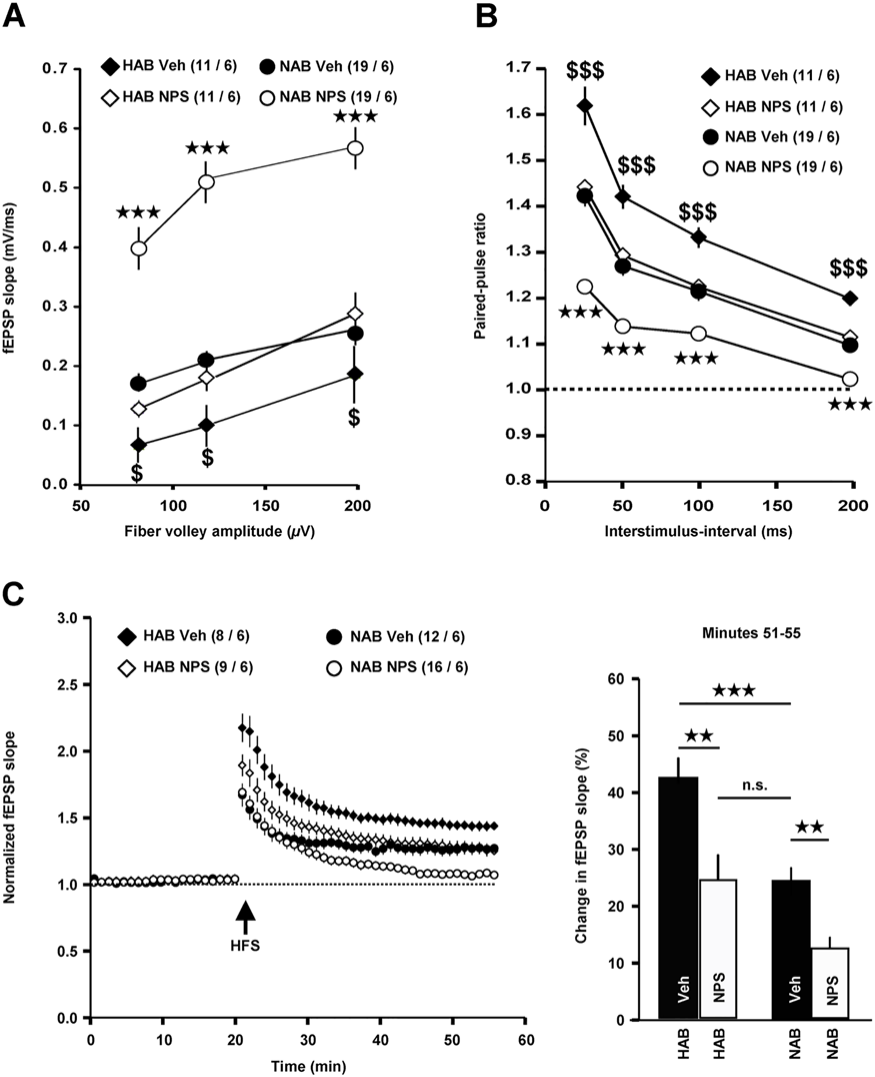
Intranasally applied NPS (open diamonds and open circles) causes enhanced basal neurotransmission (*A*) and reduced PPF (*B*) and LTP (*C*) at ventral CA3-CA1 synapses in HAB and NAB mice compared to Veh-treated (black diamonds and black circles) animals. *n* numbers are indicated in brackets as follows (slices / animals). ★ Comparison NAB Veh *vs*. NAB NPS. $ Comparison HAB Veh *vs*. HAB NPS.

Concerning basal neurotransmission (Fig. 3*A*), three-way ANOVA comparison of the 4 experimental groups showed a significant effect of the Line (F_1,56_ = 63.561, *p* < 0.001), of the Treatment (F_1,56_ = 55.409, *p* < 0.001), and a significant interaction Line x Treatment (F_1,56_ = 16.76, *p* < 0.001), but no significant interaction Line x Treatment x FVA (F_2,112_ = 1.606, *p* = 0.205), suggesting that the NPS effect is independent of the FVA. Group comparison revealed that Veh-treated HAB mice (*n* = 11 slices / 6 animals) showed a significantly weaker basal neurotransmission compared to Veh-treated NAB mice (*n* = 19 slices / 6 animals) (Tukey *post hoc* tests: 80 *μ*V FVA, *p* = 0.006; 120 *μ*V FVA, *p* = 0.003; 200 *μ*V FVA, *p* = 0.049) and that intranasal application of NPS increased basal neurotransmission both in HAB (*n* = 11 slices / 6 animals) and NAB (*n* = 19 slices / 6 animals) mice (one-way ANOVA: F_3,59_ > 35.49, *p* < 0.001). Interestingly, basal neurotransmission in NPS-treated HAB mice is not significantly different from that in Veh-treated NAB animals (Tukey *post hoc* tests: 80 *μ*V FVA, *p* = 0.239; 120 *μ*V FVA, *p* = 0.415; 200 *μ*V FVA, *p* = 0.388).

Concerning short-term plasticity (PPF, Fig. 3*B*), three-way ANOVA comparison of the 4 experimental groups showed a significant effect of the Line (F_1,51_ = 74.04, *p* < 0.001), of the Treatment (F_1,51_ = 54.78, *p* < 0.001), but no significant interaction Line x Treatment (F_1,51_ = 0.001, *p* = 0.978) or Line x Treatment x ISI (F_3,153_ = 1.283, *p* = 0.282), suggesting that the NPS effect is independent of the ISI. Group comparison revealed that Veh-treated HAB mice exhibited a higher PPF compared to Veh-treated NAB mice (Tukey *post hoc* tests: ISI 25, 50, 100, and 200 ms, *p* < 0.001) and that both Veh-treated groups showed a significantly higher PPF compared to their NPS-treated counterparts (one-way ANOVA: F_3,54_ > 26.96, *p* < 0.001). Again, PPF in NPS-treated HAB mice is not significantly different from that in Veh-treated NAB animals (Tukey *post hoc* tests: ISI 25 ms, *p* = 0.816; ISI 50 ms, *p* = 0.806; ISI 100 ms, *p* = 0.976; ISI 200 ms, *p* = 0.756).

Finally, LTP magnitude was also different between the 4 groups (one-way ANOVA: F_3,41_ = 18.56, *p* < 0.001). As could be expected from the data shown in Fig. 2*C*, Veh-treated HAB mice (*n* = 8 slices / 6 animals) exhibited a higher LTP magnitude compared to Veh-treated NAB mice (*n* = 12 slices / 6 animals) (Fig. 3*C*, LTP minutes 51–55: Tukey *post hoc* tests, *p* < 0.001). In both lines, intranasal application of NPS produced a decrease in LTP magnitude (Fig. 3*C*, LTP minutes 51–55: NAB Veh *vs*. NAB NPS (*n* = 16 slices / 6 mice), *p* = 0.006; HAB Veh *vs*. HAB NPS (*n* = 9 slices / 6 animals), *p* = 0.002). Again, LTP magnitude between NPS-treated HAB and Veh-treated NAB mice is not significantly different (Fig. 3*C*, *p* = 0.998).

Taken together, our results demonstrate that intranasal application of NPS induces similar effects both in HAB and NAB mice: increased basal neurotransmission and decreased short-term and long-term plasticity. Even more interestingly, intranasal NPS treatment shifts the electrophysiological endophenotype present in HAB mice towards a “normal”-anxiety one.

## Discussion

Here, we uncovered a multifaceted high-anxiety electrophysiological endophenotype in the murine vHPC and provide the first experimental evidence that an intranasally applied neuropeptide can shift such an endophenotype in an anxiety-regulating brain structure towards a “normal”-anxiety one. In particular, using electrophysiological techniques in acute hippocampal slices, we show that basal neurotransmission at ventral CA3-CA1 synapses is weaker in HAB compared to NAB mice. We further revealed that, at these synapses, PPF and LTP are more pronounced in slices from HAB animals. Remarkably, in slices from HAB mice which were intranasally treated with NPS, we detected enhanced basal neurotransmission and reduced PPF and LTP.

The decreased magnitude of PPF suggests that NPS led to an activation of presynaptic NPSRs [24], as it has been demonstrated to occur at glutamatergic synapses onto intercalated GABAergic neurons in the amygdala [12]. Consistently, we previously found that administration of NPS to murine vHPC slices diminishes PPF at CA3-CA1 synapses in a NPSR-dependent manner [13]. The reduced LTP, however, argues for an additional activation of postsynaptic NPSRs [25]. This scenario is further supported by the observations that CA1 pyramidal cells take up intranasally administered Cy3-NPS and that application of NPS to mouse vHPC slices decreases CA1 LTP in a NPSR-dependent manner [13]. These long-lasting effects of NPS after intranasal administration are in accordance with previous studies in mice [13] and in rats [14], which showed behavioral effects of NPS up to 4 h after intranasal application. This can be explained by the fact that the NPSR is a GPCR and, thus, the binding of its ligand will activate the downstream signaling pathways responsible for the observed long-lasting changes, independently of whether this ligand is continuously present or washed out or metabolized during the time course of the experiments.

The combination of the following facts and findings strongly suggests that the high-anxiety endophenotype in HAB mice is indeed causally related to the extremely anxious behavior of these animals (Fig. 1, [21], [23], [26]). First, ventral CA1 pyramidal neurons, which become excited by neurotransmission at CA3-CA1 synapses, are part of a distributed neuronal circuit that participates in the control of anxiety in mice [11]. Second, the vHPC is significantly involved in the expression of anxiety-related behaviors on the EPM [5], [9]. Third, application of NPS to murine vHPC slices reduces PPF and LTP at CA3-CA1 synapses [13]. Fourth, microinjections of NPS into the ventral CA1 region cause an anxiolytic-like effect in mice [20]. And fifth, the altered neurophysiological parameters in slices from NPS-treated HAB mice (Fig. 3) were detected in a time window where such animals display decreased anxiety-related behavior [13].

How might the high-anxiety endophenotype in the vHPC of HAB mice contribute to their hyper-anxious behavior? A possible answer to this question relies on studies showing that ventral CA1 pyramidal cells directly or indirectly drive neuronal activity in the amygdala, the medial prefrontal cortex, and the bed nucleus of the stria terminalis (for review see [27]). In turn, increased neuronal activity in these brain structures has been repeatedly demonstrated to be associated with enhanced anxiety-related behavior [8–9], [28–32]. Therefore, it seems likely that anxiety-related behavior in mice and other mammals is partly regulated, in a positively correlative manner, by the firing activity of ventral CA1 pyramidal cells. Convincing evidence for this scenario is yielded by a recent report showing that optogenetic activation of excitatory projections from the basolateral amygdala to ventral CA1 pyramidal neurons gives rise to increased anxiety levels in mice [11]. Thus, stronger neurotransmission at ventral CA3-CA1 synapses would probably promote exaggerated anxiety as seen in HAB animals. While the more pronounced PPF and LTP in slices from HAB mice (Fig. 2) speak in favor of this scenario, the weaker basal neurotransmission is, at first glance, contradictory. Yet, *in vivo* recordings from ventral CA3 pyramidal cells revealed that these neurons exhibit discharge activities which should result in intense PPF or frequency facilitation at CA3-CA1 synapses [33–35]. Hence, it is tempting to speculate that the more pronounced PPF and LTP in HAB mice override the weaker basal neurotransmission, thereby leading to a stronger net excitation of ventral CA1 pyramidal cells in these animals.

To summarize, our work provides the first experimental link between pathologically high anxiety-related behavior [36] and “abnormal” functional properties of a glutamatergic vHPC synapse. The finding that intranasally delivered NPS can largely “normalize” these properties further underscores this neuropeptide as a promising novel treatment option for pathologically high anxiety.
